# The mechanosensitive Piezo1 channels contribute to the arterial medial calcification

**DOI:** 10.3389/fphys.2022.1037230

**Published:** 2022-11-10

**Authors:** László Szabó, Norbert Balogh, Andrea Tóth, Ágnes Angyal, Mónika Gönczi, Dávid Máté Csiki, Csaba Tóth, Ildikó Balatoni, Viktória Jeney, László Csernoch, Beatrix Dienes

**Affiliations:** ^1^ Department of Physiology, Faculty of Medicine, University of Debrecen, Debrecen, Hungary; ^2^ Doctoral School of Molecular Medicine, Faculty of Medicine, University of Debrecen, Debrecen, Hungary; ^3^ ELKH-DE Cell Physiology Research Group, University of Debrecen, Debrecen, Hungary; ^4^ MTA-DE Lendület Vascular Pathophysiology Research Group, Research Centre for Molecular Medicine, Faculty of Medicine, University of Debrecen, Debrecen, Hungary; ^5^ Doctoral School of Molecular Cell and Immune Biology, Faculty of Medicine, University of Debrecen, Debrecen, Hungary; ^6^ Department of Surgery, Faculty of Medicine, University of Debrecen, Debrecen, Hungary; ^7^ Clinical Center, University of Debrecen, Debrecen, Hungary

**Keywords:** vascular smooth muscle, mechanosensation, Piezo1, calcification, intracellular calcium concentration, Yoda1, Dooku1

## Abstract

Vascular calcification (VC) is associated with a number of cardiovascular diseases, as well as chronic kidney disease. The role of smooth muscle cells (SMC) has already been widely explored in VC, as has the role of intracellular Ca^2+^ in regulating SMC function. Increased intracellular calcium concentration ([Ca^2+^]_i_) in vascular SMC has been proposed to stimulate VC. However, the contribution of the non-selective Piezo1 mechanosensitive cation channels to the elevation of [Ca^2+^]_i_, and consequently to the process of VC has never been examined. In this work the essential contribution of Piezo1 channels to arterial medial calcification is demonstrated. The presence of Piezo1 was proved on human aortic smooth muscle samples using immunohistochemistry. Quantitative PCR and Western blot analysis confirmed the expression of the channel on the human aortic smooth muscle cell line (HAoSMC). Functional measurements were done on HAoSMC under control and calcifying condition. Calcification was induced by supplementing the growth medium with inorganic phosphate (1.5 mmol/L, pH 7.4) and calcium (CaCl_2_, 0.6 mmol/L) for 7 days. Measurement of [Ca^2+^]_i_ using fluorescent Fura-2 dye upon stimulation of Piezo1 channels (either by hypoosmolarity, or Yoda1) demonstrated significantly higher calcium transients in calcified as compared to control HAoSMCs. The expression of mechanosensitive Piezo1 channel is augmented in calcified arterial SMCs leading to a higher calcium influx upon stimulation. Activation of the channel by Yoda1 (10 μmol/L) enhanced calcification of HAoSMCs, while Dooku1, which antagonizes the effect of Yoda1, reduced this amplification. Application of Dooku1 alone inhibited the calcification. Knockdown of Piezo1 by siRNA suppressed the calcification evoked by Yoda1 under calcifying conditions. Our results demonstrate the pivotal role of Piezo1 channels in arterial medial calcification.

## 1 Introduction

Vascular calcification (VC) is defined as a deposition of calcium-phosphate complexes in large and medium sized blood vessels. The process is highly correlated with several cardiovascular diseases, depending on the organs affected and the degree of progress.

Mineral deposition can affect both the intimal and medial layers of blood vessels. Calcification occurring in the intimal layer of arteries (AIC), which is normally composed of endothelial cells and subendothelial connective tissue, is associated with atherosclerotic lesions (Sangiorgi et al., 1998; Hunt et al., 2002) with further risk of obstructive vascular diseases: unstable and rupturable plaque formation (Hutcheson et al., 2016), and myocardial infarction (Ehara et al., 2004) or stroke (Vliegenthart et al., 2002). Several mechanisms have been documented as known mediators of this process: lipid deposition, monocyte/macrophage infiltration, mitochondrial dysfunction and oxidative stress, mechanical stress, migration and proliferation of SMCs, and dysfunction of extracellular matrix (ECM) proteins, in response to chronic arterial inflammation and SMC apoptosis (Doherty et al., 2003; Chelu et al., 2006; Durham et al., 2018).

The calcification of the medial layer (AMC (arterial medial calcification), or Monckeberg’s sclerosis), which consists of SMCs and the elastin-rich extracellular matrix, is not necessarily associated with lipid accumulation and inflammatory cell invasion, but other factors mentioned above in AIC are known drivers of AMC as well. Furthermore, cell senescence is also suggested to initiate AMC. AMC leads to vascular stiffness, systolic hypertension and increases the prevalence of diastolic dysfunction, increased afterload, and heart failure (Essalihi et al., 2004; Lanzer et al., 2014; Chow and Rabkin, 2015). Although aging is considered as physiological trigger for AMC, certain pathological conditions such as chronic kidney disease (CKD), diabetes mellitus, hypertension, D hypervitaminosis, or rare hereditary disorders are known to hasten the process (Wu et al., 2013).

Whichever case is examined, partially overlapping mechanisms that were proposed to induce calcification all implicate the involvement of SMCs of the medial layer. It is now generally accepted that VC, although previously thought to be a passive, is an active process whose regulation is highly dependent on the SMCs. While the contractile phenotype contributes significantly to the physiological function of the cardiovascular system, and the maintenance and remodelling of the extracellular matrix, upon pathological signals the phenotypic plasticity of SMCs makes them capable of transforming into other phenotypes including adipocytic, senescent, foam cell, synthetic and osteochondrogenic (Durham et al., 2018), which are conducive to vascular calcification. The osteochondrogenic transdifferentiation of vascular SMCs has a central role in coordinating the processes leading to VC (Shanahan et al., 1999). In parallel with this phenotypic change, calcifying extracellular vesicles deposited and aggregated in the vessel wall are also secreted by the SMCs (Reynolds et al., 2004; Rodenbeck et al., 2017; Schurgers et al., 2018). Under physiological conditions, these vesicles support to maintain homeostasis and do not calcify. They contain calcification inhibitors, as matrix GLA protein and fetuin-A (Reynolds et al., 2004; Kapustin et al., 2015). Loss of these inhibitors increases the probability of the appearance of nucleation sites of mineralization (Reynolds et al., 2004; Reynolds et al., 2005; Schurgers et al., 2018). These phenomena resemble bone mineralization (Shanahan et al., 2011), since in growth plate chondrocytes, an intracellular signal has been proposed to initiate transdifferentiation and release of calcifying matrix vesicles (Wang and Kirsch, 2002). During VC, the effect of increased extra- or intracellular Ca^2+^ levels have also been widely studied, and a clear connection to the calcification has been documented (Fleckenstein-Grün et al., 1992; Olszak et al., 2000; Shroff et al., 2008). Several studies have reported the role of elevated extracellular Ca^2+^ level in SMCs differentiation (Chang et al., 2002; Wu et al., 2003; Reynolds et al., 2004; Yang et al., 2004). A recent study of Kapustin et al. (2011) assigned the mineralization of matrix vesicles to disturbed intracellular calcium homeostasis, which results in inhibitor depletion and the formation of Annexin 6/phosphatidylserine nucleation complexes. However, the mechanism whereby the intracellular calcium level is increased is not properly understood.

Since the identification of Piezo channels over the past decade (Coste et al., 2010), a number of studies have demonstrated their role in vascular physiology. These transmembrane proteins function as mechanosensitive, non-selective cation channels and their contribution to the altered intracellular calcium homeostasis has been shown to be significant in many cardiovascular diseases (Beech and Kalli, 2019; Douguet et al., 2019). It is known that the Piezo1 channels in the endothelium of blood vessels play an essential role in the early development of the vascular system and the lack of the channel or a loss of function mutation at the embryonic stage is lethal due to the disorganized vascular structure (Li et al., 2014; Ranade et al., 2014). Expression of Piezo1 is high in SMCs of resistance arteries as well. Although Piezo1 seems to be dispensable for myogenic tone induced by an increase in wall tension, the channel is essential in the arterial remodelling of arteries during hypertension (Retailleau et al., 2015). Calcium influx through Piezo1 leads to an increase in intracellular Ca^2+^ concentration. It has already been reported that the elevated cytosolic Ca^2+^ concentration stimulates transglutaminase II activity, resulting in the remodelling of the cytoskeleton, as well as of the extracellular matrix (Retailleau et al., 2015). This pathway links the Piezo1 channel opening of arterial SMCs to systemic hypertension. A recently published study of Liao et al. (2021) explores the connection between Piezo1 upregulation and altered Ca^2+^ homeostasis of pulmonary arterial SMCs resulting in a hyperproliferative and hypercontractive phenotype under pulmonary arterial hypertension conditions.

Here we propose that the mechanosensitive Piezo1 channels expressed on the SMCs of large systemic arteries have pivotal role in VC. They are overexpressed on calcifying SMCs, and, furthermore, their activation augments, while their inhibition or the downregulation of their expression hinders AMC.

## 2 Materials and methods

### 2.1 Cell culture and treatments

Human aortic SMCs (HAoSMC; 354-05; Cell Applications Inc., San Diego, CA, United States) were maintained in growth medium (GM) that was prepared by supplementing Dulbecco’s Modified Eagle Medium (DMEM, D6171, Sigma) with 10% FBS (10270-106, Gibco, Grand Island, NY, United States), antibiotic antimycotic solution (A5955, Sigma), sodium pyruvate (S8636, Sigma) and L-glutamine (G7513, Sigma). Cells were maintained at 37°C in a humidified atmosphere containing 5% CO_2_. Cells were grown to confluence and used from passages 5 to 8.

To induce calcification we cultured VSMCs in osteogenic medium (OM) that was obtained by supplementing GM with inorganic phosphate (Pi) (NaH_2_PO_4_-Na_2_HPO_4_, 1.5 mmol/L, pH 7.4) and calcium (CaCl_2_, 0.6 mmol/L). A stock solution of Yoda1 (50 mmol/L) was prepared in DMSO. In calcification experiments Yoda1 was used at a concentration of 10 μmol/L and DMSO was used as a vehicle control. Yoda1 and Dooku1 were co-incubated for 5 min before changing the media every 2 days (Villa-Bellosta and Egido, 2017).

### 2.2 Human samples

Human internal carotid artery, and superficial femoral artery samples were obtained from vascular surgical interventions. The samples were collected, stored and handled based on and in accordance with the permit number DE RKEB/IKEB 4916-2017 issued by Regional and Institutional Research Ethics Committee of the Clinical Center of the University of Debrecen.

### 2.3 Isolation of smooth muscle cells from mouse abdominal artery

Vascular smooth muscle cells were obtained from adult (24-week-old) C57BL6 mice. Mice were euthanized by isoflurane then placed in supine position under a dissecting microscope. The skin was removed from the thorax and abdomen. Thorax was opened and the aorta was perfused with sterile PBS. Abdominal aorta was removed from the heart to the iliac bifurcation. Adventitia was removed from aorta. The smooth muscle layer of the aorta was cut into approximately 1 × 1 mm pieces. The smooth muscle tissue was treated with collagenase type II (C-6885, Sigma, Burlington, MA, United States) solution at 37 C, 5% CO_2_ for 5 hours. The cells were centrifuged for 5 min, 300 ×*g* at room temperature and resuspended in PBS to stop the enzyme. All experimental procedures with animals were performed in accordance with the Institutional Guidelines for the Care and Use of Laboratory Animals.

### 2.4 Immunolabeling, confocal imaging

Freshly isolated mouse aortic smooth muscle cells and HAoSMCs were seeded onto glass coverslips and kept at 37°C for 1–2 h while attached to the surface of the coverslips. After fixed in 4% PFA and repeated washing in glicine-PBS solution (100 mM, 15 min, 22°C), cells were permeabilized with Triton-X-100 (0.5 v/v%, 10 min, 22°C). After PBS washing (3x) non-specific biding sites were blocked with Carbo-Free Blocking Solution (SP-5040, VECTOR Laboratories, Burlingame, CA, United States). Immunostaining was performed using anti-Piezo1 (Thermo Fisher Scientific, Rockford, IL, United States, MA5-32876, mouse-IgG, monoclonal) primary antibody with Cy3 anti-mouse secondary antibody labelling (A10521, Life technologies, Eugene, OR, United States). Images were taken using Zeiss laser scanning confocal microscope (Zeiss LSM880 Airyscan; Zeiss, Oberkochen, Germany) using 405, 488 and 543 nm excitation wavelengths and 10x or 63x oil immersion objective. On HAoSMCs anti-Piezo1 and anti-alfa smooth muscle specific actin (αSMA) (Thermo Fisher Scientific, Rockford, IL, United States, MA5-32876, mouse-IgG, monoclonal; PA516697, rabbit-IgG, polyclonal, respectively) primary antibodies were applied.

Paraffin embedded human aorta samples were subjected to deparaffinization process, and the protocol described above was applied as staining procedure.

### 2.5 Quantitative real time polymerase chain reaction for Piezo1

Q-PCR was performed on Stratagene Mx3005P QPCR System from Agilent Technologies (Santa Clara, California, United States) using the 5′ nuclease assay. Total RNA was isolated using TRIzol from LifeTechnologies (Carlsbad, California, United States), DNase 5 treatment was performed according to the manufacturer’s protocol, and then 1 μg of total RNA were transcribed into cDNA using 15 IU of AMV reverse transcriptase (4368814, Thermo Fisher Scientific, Rockford, IL, United States). PCR amplification was performed using the TaqMan primers and probes and the TaqMan universal PCR master mix protocol (4369016, Thermo Fisher Scientific, Rockford, IL, United States). As internal control transcripts of Peptidylprolyl isomerase—A (PPIA) (Hs99999904_m1, Thermo Fisher Scientific, Rockford, IL, United States) was applied each cases. Piezo1 gene mRNA expression was measured using PIEZO1 primers (Hs00207230_m1, Thermo Fisher Scientific, Rockford, IL, United States). The amount of the transcripts was normalized at first, to the relevant housekeeping gene using the ΔCT method. The final results were then normalized to the expression of the control or scrambled samples (ΔΔCT method).

### 2.6. Real**-**time qPCR for transglutaminase 2

RNA from human aortic VSMCs was isolated using TRIzol (CS502, RNA-STAT60, Tel-Test Inc.; Friendswood, TX, United States) followed by cDNA synthesis using High-Capacity cDNA Reverse Transcription Kit (4368813, Applied Biosystems, Waltman, MA, United States). The qPCR was performed in triplicate using LightCycler 480 SYBR GREEN I Master Mix (04887352001, Roche Diagnostics GmbH, Mannheim, Germany). Reactions were performed in a Real Time PCR System (Bio-Rad Laboratories, Hercules, CA, United States). Primers used are as follows: forward (5′-TGC​TGG​GCC​ATT​CAT​TTT​G-3′) and reverse (5′-TCT​TCC​GAG​TCC​AGG​TAC​AC-3′). Relative mRNA expressions were calculated with the ∆∆Ct method, using HPRT as internal control.

### 2.7 Western blotting

For Western blot experiments total cell lysates were isolated from control, calcified and gene silenced HAoSMC cell lines. The samples were subjugated to sonication. Protein concentration were measured. Samples were subjected to SDS-PAGE (10% gels loaded with 20 μg protein per lane) and transferred to nitrocellulose membranes (Bio-Rad). The protein-binding nitrocellulose membranes were then blocked with 5% dry milk-PBS solution. Proteins were probed with anti-Piezo1 (Thermo Fisher Scientific, Rockford, IL, United States, MA5-32876, mouse-IgG, monoclonal) or with anti-Runx2 (20700-1-AP; Proteintech, Rosemont, IL) primary antibodies followed by HRP-conjugated secondary antibody labeling. The primary-secondary antibody complexes were detected using an enhanced chemiluminescence Western blotting Pico or Femto kit (Thermo Scientific, Rockford, IL, United States) in a Fujifilm Labs-3000 dark box. According to the manufacturers’ descriptions, the band corresponding to the Piezo1 protein in cell lysates can be detected above 250 kDa, at ∼280–300 kDa, while that of the Runx2 protein is 57–60 kDa.

### 2.8 Measurement of intracellular Ca^2+^ concentration [Ca^2+^]_i_ in cultured cells

Cells were loaded with 10 mM Fura-2-AM (F1221, Thermo Fisher Scientific, Rockford, IL, United States) for 50 min at 37°C. After loading the cells were kept in Tyrode’s solution. Fura-2 was excited with a CoolLED pE-340^fura^ light source (CoolLED LTD., Andover, England) mounted on a ZEISS Axiovert 200 m inverted microscope (Zeiss, Oberkochen, Germany). The excitation wavelengths were alternating between 340 and 380 nm wavelength, the emission was detected with a band-pass filter 505–570 nm. The image acquisition and post processing was made with the AxioVision (rel. 4.8) software (Zeiss, Oberkochen, Germany). The [Ca^2+^]_i_ was calculated from the ratio of the images taken at 340 and 380 nm after background correction with constants determined during *in vitro* calibration of the setup. After that, the relative changes in the ratio of the two wavelengths as a result of stimulation have been analyzed.

### 2.9 Alizarin red staining and quantification

After washing with DPBS (Dulbecco’s Phosphate Buffered Saline, 14190144, Gibco, Waltham, MA, United States) the cells were fixed in 4% paraformaldehyde (16005, Sigma, Burlington, MA, United States) and rinsed with deionized water thoroughly. Cells were stained with Alizarin Red S (A5533, Sigma, Burlington, MA, United States) solution (100 mmol/L) to the wells and measured optical density (OD) at 560 nm using hexadecyl-pyridinium chloride solution as blank.

### 2.10 Quantification of calcium deposition

Cells grown on 96-well plates were washed twice with DPBS, and decalcified with HCl (30721, Sigma, 0.6 mol/L) for 30 min at room temperature. Calcium content of the HCl supernatants was determined by QuantiChrome Calcium Assay Kit (DICA-500, Gentaur, Kampenhout, Belgium). Following decalcification, cells were washed twice with DPBS, and solubilized with a solution of NaOH (S8045, Sigma, Burlington, MA, United States, 0.1 mol/L) and sodium dodecyl sulfate (11667289001, Sigma, Burlington, MA, United States, 0.1%), and protein content of samples were measured with BCA protein assay kit (23225, Pierce Biotechnology, Rockford, IL, United States). Calcium content of the cells was normalized to protein content and expressed as μg/mg protein.

### 2.11 Quantification of osteocalcin

For osteocalcin (OCN) detection, the ECM of the cells grown on 6-well plates was dissolved in 100 μl of EDTA (E6758, Sigma, 0.5 mol/L, pH 6.9). OCN content of the EDTA-solubilized ECM samples was quantified by an enzyme-linked immunosorbent assay (ELISA) (DY1419-05, DuoSet ELISA, R&D, Minneapolis, MN, United States) according to the manufacturer’s protocol.

### 2.12 *Ex vivo* aorta organ culture model and quantification of aortic calcium

C57BL/6 mice (8–12 weeks old male, n = 18) were exterminated by CO_2_ inhalation and perfused with 5 ml of sterile DPBS. The entire aorta was harvested and cleaned under aseptic conditions and cut into pieces. Aorta rings were maintained in control, OM and OM plus Yoda1 (OMY). After 7 days the aorta pieces were washed in DPBS, opened longitudinally and decalcified in 25 µl of 0.6 mmol/L HCl overnight. Calcium content was determined by QuantiChrom Ca-assay kit as described previously.

### 2.13 Histology

Aortic rings from the *ex vivo* culture model were fixed in 10% neutral buffered formalin (HT501640; Sigma, Burlington, MA, United States), embedded in paraffin blocks and cut into 4 µm thick cross-sections. After deparaffinization and rehydration, von Kossa staining and hematoxylin eosin (H&E) counterstaining on the sections were performed according to the manufacturer’s protocol (Von Kossa kit, ab150687, Abcam, Cambridge, United Kingdom).

During Masson’s trichrome staining after deparaffinization procedure and antigen retrieval Weigert´s Iron hematoxylin solution (solution A: 5 mg Hematoxylin dissolved in 500 ml 95% ethanol; Solution B: ferric chloride, distilled water, hydrochlorid acid; mixed in 1:1 ratio) was applied for 10 min then samples were rinsed in tap water (5 min) and distilled water. In the next step Biebrich Scarlet stain (Biebrich scarlet 2.7 mg, acid fuchsin 0.3 mg, distilled water 300 ml, glacial acetic acid 3 ml) was subjected (5 min) followed by rinse in distilled water. Next phosphotungstic/phosphomolybdic acid (in 1:1 ratio, dissolved in distilled water) for 10 min was used, then slides were transferred into Aniline blue (dissolved in distilled water and supplemeted with 1 V/V% Glacial acetic acid) for 5 min 1% acetic acid solution (1 min), final washing with distilled water, dehydration, clearing of the slides and application of coverslip completed the staining protocol.

### 2.14.RNA silencing

To knock-down Piezo1 gene expression we used *Silencer* ^®^ select siRNA constructs targeting Piezo1 (Cat# 4392421, Thermo Fisher Scientific). As a control negative control #1 construct (#4390843, Thermo Fisher Scientific) was used. Lipofectamine^®^ RNAiMAX reagent (13778075, Invitrogen, Carlsbad, CA, United States) was used to transfect VSMCs according to the manufacturer’s protocol.

### 2.15 Statistical analysis

Pooled data were expressed as mean ± standard error of the mean (SEM) or standard deviation (SD). The differences between statistical groups were assessed using Student’s t-test with Prism (GraphPad Software, San Diego, CA, United States) and a *p* value of less than 0.05 was considered statistically significant.

## 3 Results

### 3.1 Piezo1 channels are expressed in human vascular SMCs

**FIGURE 1:**
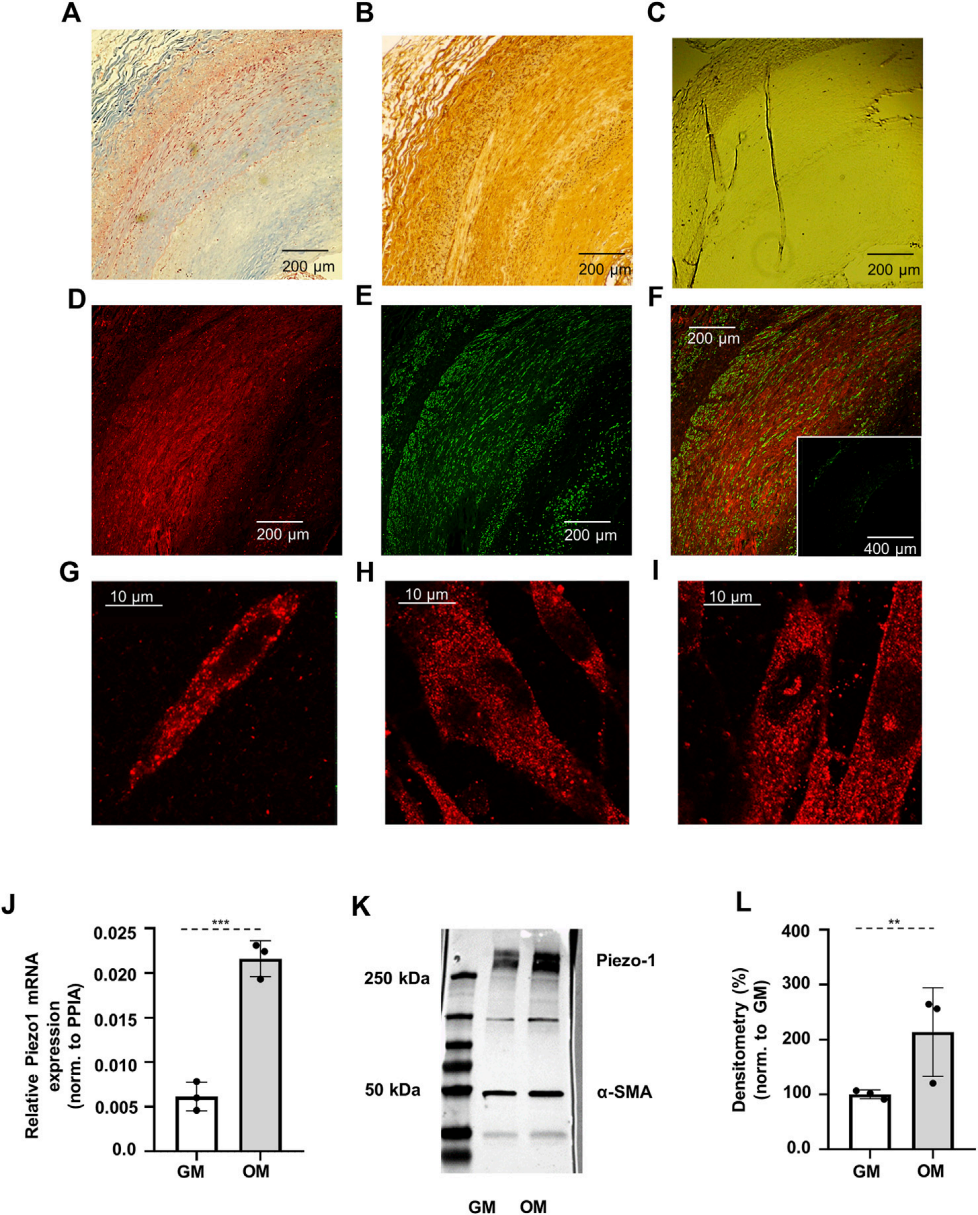
Verification of the expression of Piezo1 channels. **(A)** Masson’s trichrome staining indicates the smooth muscle layer (red color) in the sections of human internal carotid artery sample obtained from vascular surgical interventions. **(B)** DAB staining for Piezo1. The appearance of brown color in the images demonstrates the presence of Piezo1. **(C)** Negative control for DAB staining. **(D)** Localization of Piezo1 in human carotid artery marked with Cy3 (red). **(E)** Immunostaining of smooth muscle specific alfa-actin (αSMA) was used to identify smooth muscle cells in the section (green, Alexa488). **(F)** Merged immunofluorescent image from Piezo1 and αSMA. Inset: negative control of immunofluorescence labeling. **(G)** Piezo1 expression on smooth muscle cell isolated from mouse abdominal artery, and **(H–I)** on control and calcified HAoSMCs, respectively. **(J)** qPCR analysis of Piezo1 at mRNA level on HAoSMCs under control (GM) and calcifying (OM) conditions. **(K)** Representative Western blot from control (GM) and calcified (OM) HAoSMCs labelled for Piezo1 and αSMA as reference. **(L)** Semi-quantitative estimation of Piezo1 protein expression on control (GM) and calcified (OM) HAoSMCs. Results represent 3 independent experiments performed in triplicate.

Initial investigations tested the relevance of Piezo1 channels in human vascular SMCs. Human internal carotid artery, as well as superficial femoral artery samples have been examined. Immunohistochemistry of these arterial rings revealed that the Piezo1 channels are expressed in the smooth muscle layers of adult human arteries. Masson’s trichrome stain has been used to localize the smooth muscle layer in the sections, and DAB staining has been applied for the visualisation of Piezo1 channels (Figure 1A–C). Fluorescent immunohistochemical staining on successive slices confirmed these observations on human samples (Figure 1D–F).

Due to the limited availability of human samples, functional measurements were carried out on a Human Aortic Smooth Muscle Cell line (HAoSMC). In order to rule out that the observed effects are attributable to the chosen HAoSMC cell line primary SMCs freshly isolated from mouse abdominal artery were also tested in a portion of the experiments. Immunostaining of Piezo1 confirmed the presence of the channel on both cell types (Figure 1G,H). mRNA and protein expression of Piezo1 was further verified by qPCR and Western blot analysis in HAoSMCs (Figure 1J–L).

### 3.2 Calcification is accompanied by elevation of Piezo1 expression on HAoSMC

After having established that SMCs express Piezo1, alteration of its expression upon calcification was investigated. To induce calcification, HAoSMC cells were cultured in osteogenic medium (OM) (growth medium (GM) supplemented with 1.5 mmol/L phosphate and 0.6 mmol/L calcium) for 4 days, as in previous studies (Balogh et al., 2019). Following treatment, immunofluorescence assays were performed, and the expression of Piezo1 mRNA and protein in control and calcified HAoSMCs was analyzed by qPCR and Western blot, respectively. Fluorescent immunolabelling reflected the expression of the channel both on control and calcified cells (Figure 1H,I, respectively). Expression of Piezo1 mRNA was normalized to Peptidylprolyl isomerase A (PPIA), and upregulation of Piezo1 in calcified over control cells was observed (Figure 1J). Similar changes were detected at the protein level, as shown in Figure 1K, where a representative immunoblot of control and calcified HAoSMC samples is presented, while in panel **L** the quantitative densitometry analysis of three independent immunoblots is shown using α-smooth muscle actin (α-SMA) as reference. In calcified cells (OM) protein levels indicate a significant increase in the expression of Piezo1 channels, as compared to control cells (GM).

### 3.3 Influx through Piezo1 contributes to changes in intracellular calcium concentration

**FIGURE 2:**
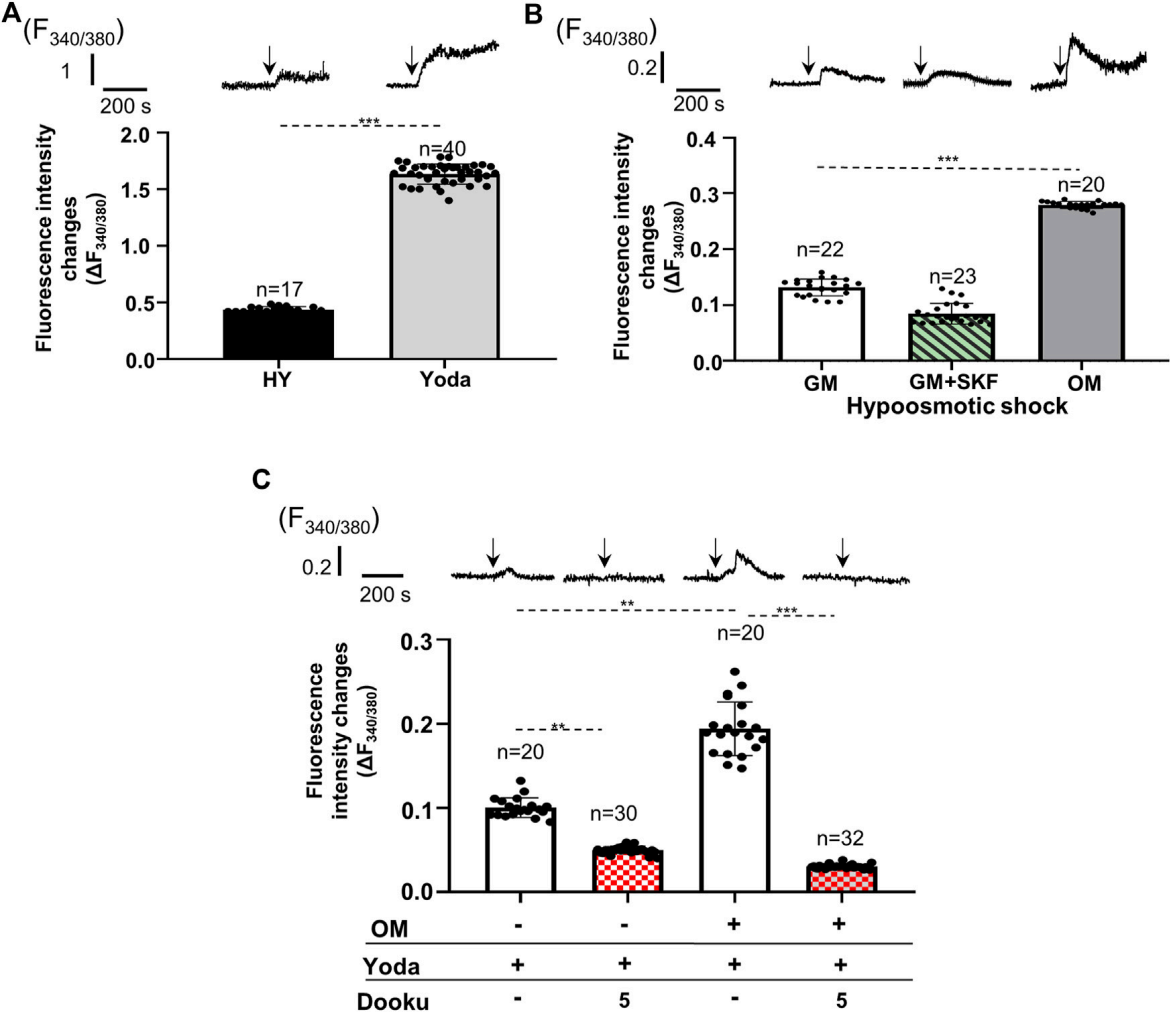
Alteration of Ca2+ transients evoked by pharmacological and mechanical activation (due to cell volume regulation) of Piezo1 channels on control and calcified smooth muscle cells. Representative transients and mean of Fura-2 fluorescence intensity changes measured on individual cells are shown. **(A)** Hypoosmotic shock (HY) and Yoda1 administration evoked Ca^2+^ transients on smooth muscle cells isolated from mouse abdominal artery. **(B)** Calcium transients elicited by hypoosmotic shock on control (GM), SKF 96365 treated (GM + SKF) and calcified (OM) HAoSMCs. **(C)** Yoda1 induced calcium transients on control (GM) and calcified (OM) HAoSMCs. Effect of additional 5 µM Dooku1, which reversibly and selectively antagonizes Yoda1 effect on Piezo1 activity is presented by patterned columns. Data are presented as mean ± SEM, **p < 0.01, ***p < 0.005. The arrows indicate the time of the appropriate intervention.

After confirming the expression of Piezo1 and changes in its expression level upon calcification, activity of the channels was examined in both cell types under different conditions. First, Piezo1 channels on SMCs isolated from mouse abdominal artery were tested. Cells were loaded with the ratiometric calcium-sensitive fluorescent dye Fura-2 and alterations in the intracellular calcium concentrations were measured using the CoolLed pE-340^fura^ illuminator mounted on a Zeiss Axioimager fluorescent microscope. Two different mechanisms were used to activate the channels: application of hypoosmotic extracellular fluid to induce mechanical stretch, and administration of Yoda1, the specific activator of Piezo1 channels. The hypoosmotic shock, as well as Yoda1 application were able to evoke calcium transients on primary SMCs isolated from mouse abdominal artery (Figure 2A). The same stimuli also resulted in calcium transients on HAoSMCs (Figures 2B,C). Since the expression of several TRP channels has been confirmed in SMCs, some of which proved to be mechanosensitive calcium channels, it was essential to rule out the possibility that the calcium transients due to hypoosmotic stimuli could be attributed exclusively to these channels. The inhibition of TRP channels by SKF 96365 (20 µM), a general TRP channel blocker, although reduced by approximately 40%, but did not eliminate the increase in intracellular calcium levels induced by the hypotonic stress on HAoSMCs (Figure 2B). These results suggested that Piezo1 channels play a central role in the formation of calcium transients in response to membrane stretch. Next, calcified (OM) HAoSMC cells have been examined. Both stimulations (hypoosmotic shock or Yoda1 application) resulted in significantly greater Ca^2+^ transients in calcified as compared to control cells (Figures 2B,C). The hypoosmotically induced membrane stretch resulted in a 2.12 ± 0.21 fold rise in the amplitude of the measured Ca^2+^ transient on calcified cells relative to the control cells. This increase could, however, partially be assigned to other stretch activated ion channels (e.g., TRPs) (Figure 2B). On the other hand, Yoda1 which is able to activate Piezo1 in the absence of mechanical signal stimulates Piezo1 channels specifically. The response to 10 μmol/L Yoda1 treatment was a 1.93 ± 0.04-fold increase of the Ca^2+^ transient amplitudes in calcified as compared to control cells (Figure 2C). Addition of 5 μmol/L Dooku1 to the extracellular environment, which reversibly and selectively antagonizes Yoda1-induced effects decreased the amplitude of the Ca^2+^ signal substantially both in control and calcified cells. The average of peak values of fluorescence intensity changes (F_340/_F_380_) stimulated by Yoda1 was reduced from 10.0 ± 1.0% to 5.0 ± 0.4% following the application of Dooku1 in control cells, while the same values in calcified cells were 19.4 ± 3.2% and 3.0 ± 0.2%, respectively, pointing out the importance of Piezo1 in the regulation of intracellular calcium concentration (Figure 2C).

### 3.4 Activation of Piezo1 increases calcification in HAoSMCs *in vitro* and in mouse aorta *ex vivo*

**FIGURE 3:**
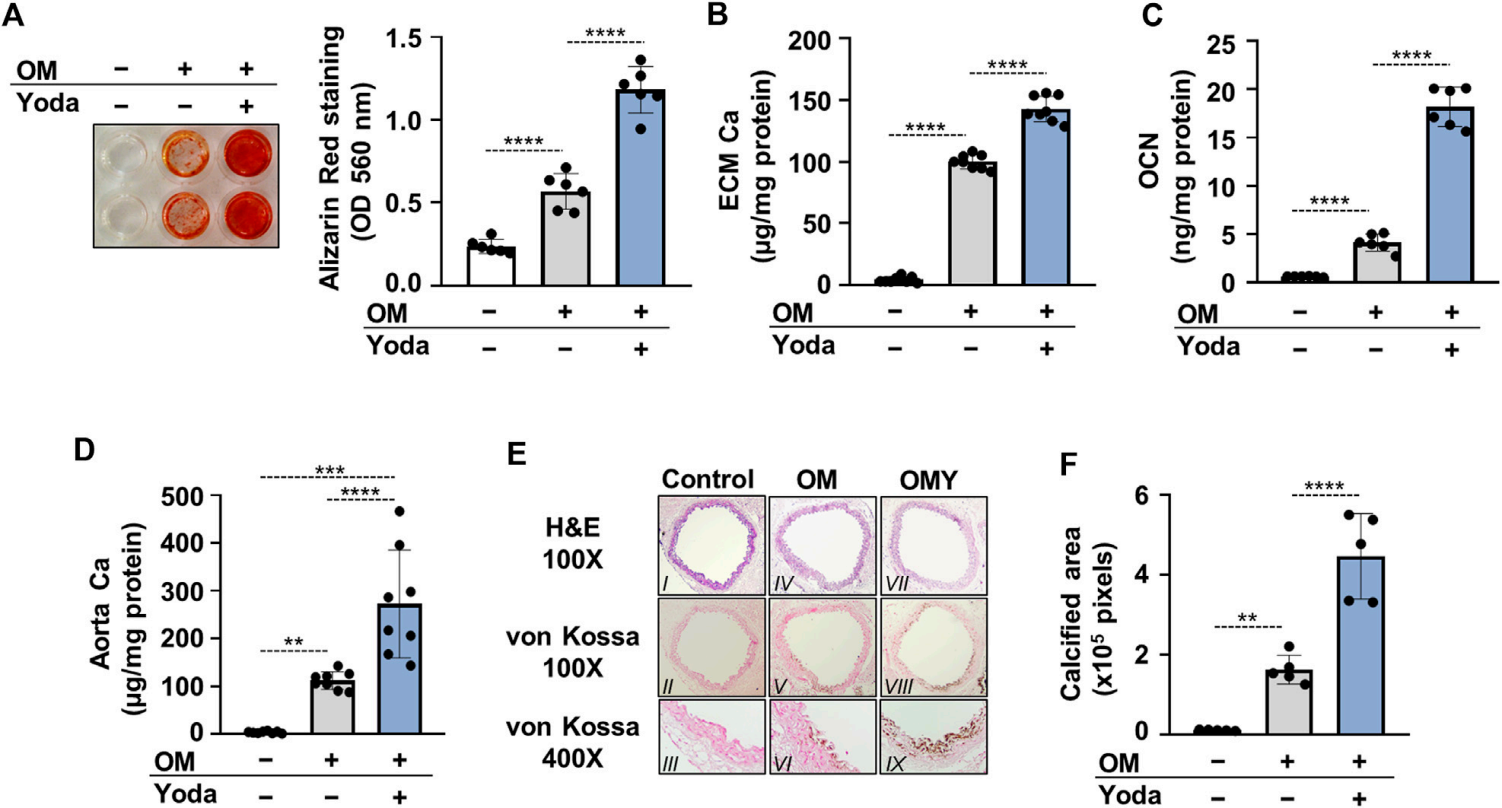
Activation of Piezo1 by Yoda1 increases Pi-mediated osteogenic differentiation and extracellular matrix (ECM) calcification of HAoSMCs and aorta rings. **(A–C)** HAoSMCs were cultured in osteogenic medium (OM; excess P_i_: 1.5 mmol/L, excess calcium: 0.6 mmol/L) in the presence or absence of Yoda1 (10 μmol/L). **(A)** Representative Alizarin Red staining (day 6) and quantification. **(B)** Calcium accumulation in the ECM (day 6). **(C)** Osteocalcin (OCN) level (day 6) in EDTA-solubilized ECM samples. **(D–E)** Aortic rings obtained from C57BL/6 mice were cultured in control, OM and OM + Yoda1 (OMY) conditions. **(D)** Calcium content of aorta rings normalized to protein level (day 6). **(E–F)** Histological analysis of *ex vivo* cultured aortic rings from C57BL/6 mice. **(E)** Representative Hematoxylin-eosin (H&E) and von Kossa–stained aortic sections of untreated, OM, and OM + Yoda1-treated (OMY) aorta rings (day 7). Magnification, ×100, ×400. **(F)** Quantification of von Kossa staining. Data are shown as mean ± SD, *n* = 3–8. *p < 0.05, **p < 0.01, ***p < 0.005, ****p < 0.001.

The influence of Piezo1 activation, and the consequently elevated intracellular calcium concentration on calcification of HAoSMCs were then addressed. Calcification of HAoSMCs was induced using an osteogenic medium (OM) in the absence or presence of Yoda1 (10 μmol/L). As revealed by Alizarin red (AR) staining and calcium measurement of the extracellular matrix (ECM) after 6 days of treatment Yoda1 largely amplified OM-induced calcification of HAoSMCs (Figures 3A,B). The level of osteocalcin (OCN), the major non-collagenous protein in bone, and a well-established marker of osteogenic differentiation of SMCs was also measured. OM induced an increase in OCN level of ECM compared to control (3.63 ± 0.64 vs 0.59 ± 0.06 ng/mg protein, respectively) which increase was further intensified by Yoda1 (18.16 ± 2.16 ng/mg protein) (Figure 3C).

For further confirmation, an *ex vivo* tissue culture model was set up and the effect of Yoda1 on aorta calcification was investigated. Cleaned aorta sections of C57BL/6 mice were cultured under control, OM and OM plus Yoda1 (OMY) conditions, and Ca^2+^ levels of aorta rings were measured on day 6. OM, as expected, increased the Ca^2+^ content of the aorta over the control, whereas the presence of Yoda1 further potentiated calcium accumulation in the aorta over OM (Figure 3D). After 7 days of culturing von Kossa staining was performed on the specimens of the descending thoracic aorta to visualize calcification. The staining revealed no calcification in control, mild calcification in OM-treated, and extensive calcification in OM plus Yoda1-treated aorta sections (Figure 3E,F) reflecting to the involvement of Piezo1 in medial calcification.

**FIGURE 4:**
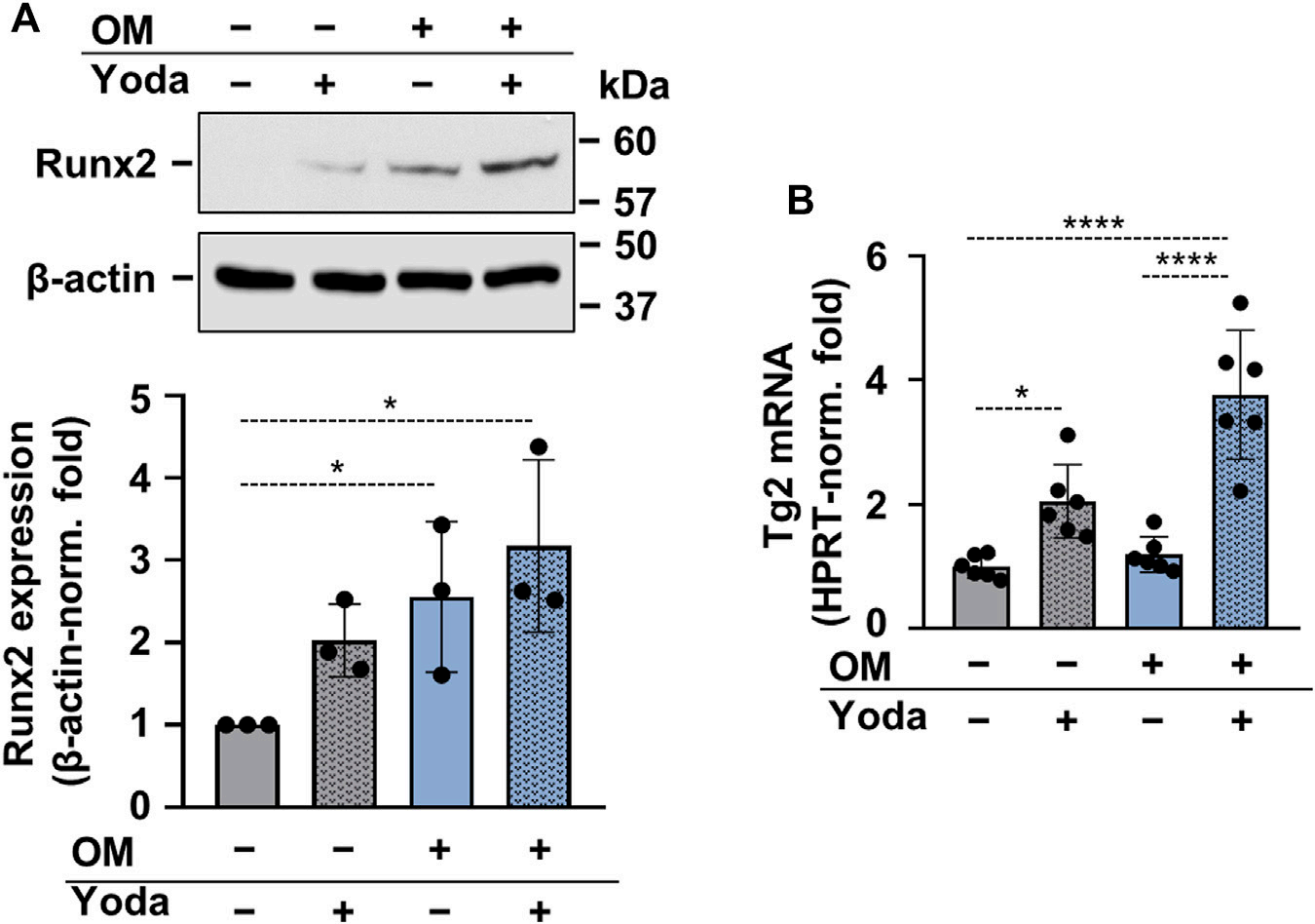
Upregulation of Runx2 and Transglutaminase 2 (Tg2) in calcifying HAoSMCs **(A,B)** HAoSMCs were exposed to Yoda1 (10 μmol/L), OM (OM; excess P_i_: 1.5 mmol/L, excess calcium: 0.6 mmol/L) or OM + Yoda1. **(A)** Protein expressions of Runx2 and β-actin were determined from whole-cell lysates (12 h). Representative Western blots out of three. β-actin-normalized relative expression of Runx2. **(B)** Tg2 mRNA levels were determined by quantitative RT-qPCR using HPRT as internal control (day 6). Data are presented as mean ± SD. *p < 0.05, ****p < 0.001.

To address whether osteogenic differentiation of HAoSMCs occurred upon stimulation with Yoda1, OM or OMY, the expression of Runx2 protein, the master transcription factor of osteogenesis was determined. Runx2 was found to be upregulated in HAoSMCs stimulated with OM and OMY as compared to control cells (Figure 4A). As Yoda1-induced Piezo1 activation had been shown to modulate the expression of transglutaminase 2 (TG2) (Retailleau et al., 2015), and TG2 plays a critical role in vascular calcification (Johnson et al., 2008), next TG2 mRNA expression of Yoda1-, OM- and OMY-treated cells were compared to control HAoSMCs. Yoda1 was found to increase TG2 mRNA level under both control and osteogenic conditions (Figure 4B).

### 3.5 Inhibition of Piezo1 activity decreases phosphate-mediated calcification in HAoSMCs *in vitro*

**FIGURE 5:**
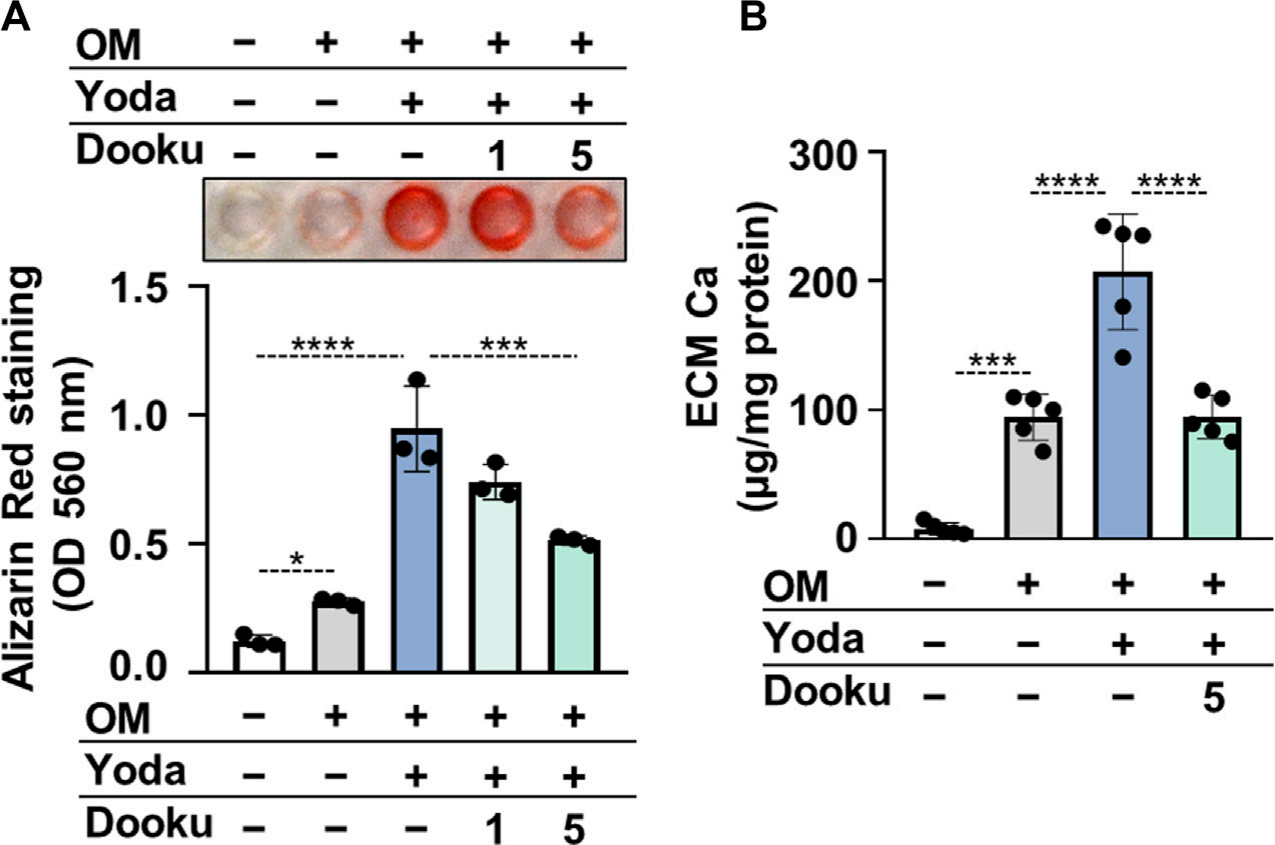
Dooku1 inhibits Yoda1-induced increase in HAoSMCs calcification. **(A,B)** HAoSMCs were exposed to OM (OM; excess P_i_: 1.5 mmol/L, excess calcium: 0.6 mmol/L) in the presence or absence of Yoda1 (10 μmol/L) and Dooku1 (1 or 5 μmol/L). **(A)** Representative Alizarin Red staining (day 6) and quantification. **(B)** Calcium content of HCl-solubilized ECM (day 6). Data are presented as mean ± SD, *n* = 3–5. *p < 0.05, ***p < 0.005, ****p < 0.001.

Next, Dooku1, an antagonist of Yoda1-evoked Piezo1 channel activity was tested to confirm the role of Piezo1 in Yoda1-induced calcification of HAoSMCs. As revealed by AR staining and calcium measurement of the ECM Dooku1 at a concentration of 5 μmol/L inhibited OMY-induced calcification of HAoSMCs (Figure 5A,B).

### 3.6 Piezo1 is indispensable to the phosphate-mediated calcification

**FIGURE 6:**
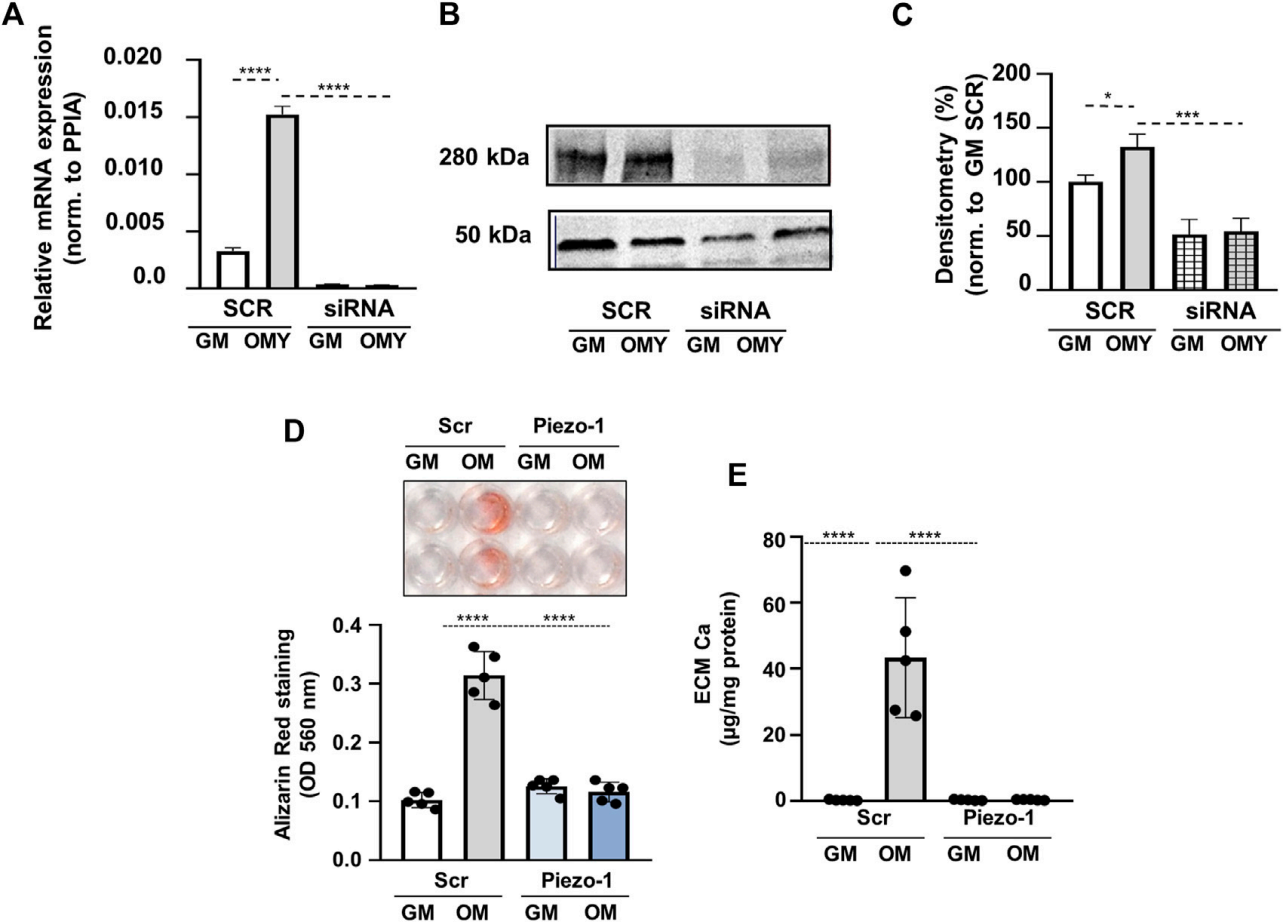
Piezo1 knockdown inhibits Yoda1-induced increase in HAoSMCs calcification. **(A–E)** HAoSMCs were exposed to control medium (GM) or OM plus Yoda1 (OMY; excess P_i_: 1.5 mmol/L, excess calcium: 0.6 mmol/L, Yoda1: 10 μmol/L) in the presence of Piezo1 targeted (siRNA) or scrambled siRNA (SCR). **(A)** Piezo1 expression at mRNA level acquired by qPCR under the aforementioned conditions. **(B–C)** Protein expression of Piezo1 in whole cell lysates (24 h). Membranes were reprobed for β-actin. Representative Western blots and relative expression of Piezo1 normalized to β-actin, respectively. **(D)** Representative Alizarin Red staining (day 4) and quantification. **(E)** Calcium content of HCl-solubilized ECM (day 4). Data are shown as mean ± SD, *n* = 5–6. ***p < 0.005, ****p < 0.001.

To further confirm the role of Piezo1 in OMY-induced calcification, Piezo1 expression has been downregulated by siRNA in HAoSMC cells (KD cells). Quantitative PCR analysis indicated significantly decreased expression of Piezo1 at mRNA level (Figure 6A), while Western blot analysis revealed the same effect on the protein level (Figures 6B,C).

Knockdown of Piezo1 by siRNA attenuated OMY-induced VSMCs calcification as detected by AR staining, as well as by the calcium content of the ECM (Figure 6D,E). AR staining in cells treated with scrambled siRNA (SC) indicated a significant increase from 0.169 ± 0.025 in GM to 0.394 ± 0.083 (*p* < 0.001) in OM complemented with 10 μmol/L Yoda1. The same values in KD cells were 0.177 ± 0.014 in GM, and 0.210 ± 0.038 in OMY. The calcium content of the extracellular matrix in SC cells was augmented from 0.28 ± 0.21 μg/mg protein in GM to 62.2 ± 21.3 μg/mg protein upon induced calcification and by additional stimulation of Piezo1. In KD cells the effect of stimulations on AR staining was missing. This dramatic suppression of calcification would only be attributed to the absence of Piezo1 channels (Figures 6D,E).

**FIGURE 7:**
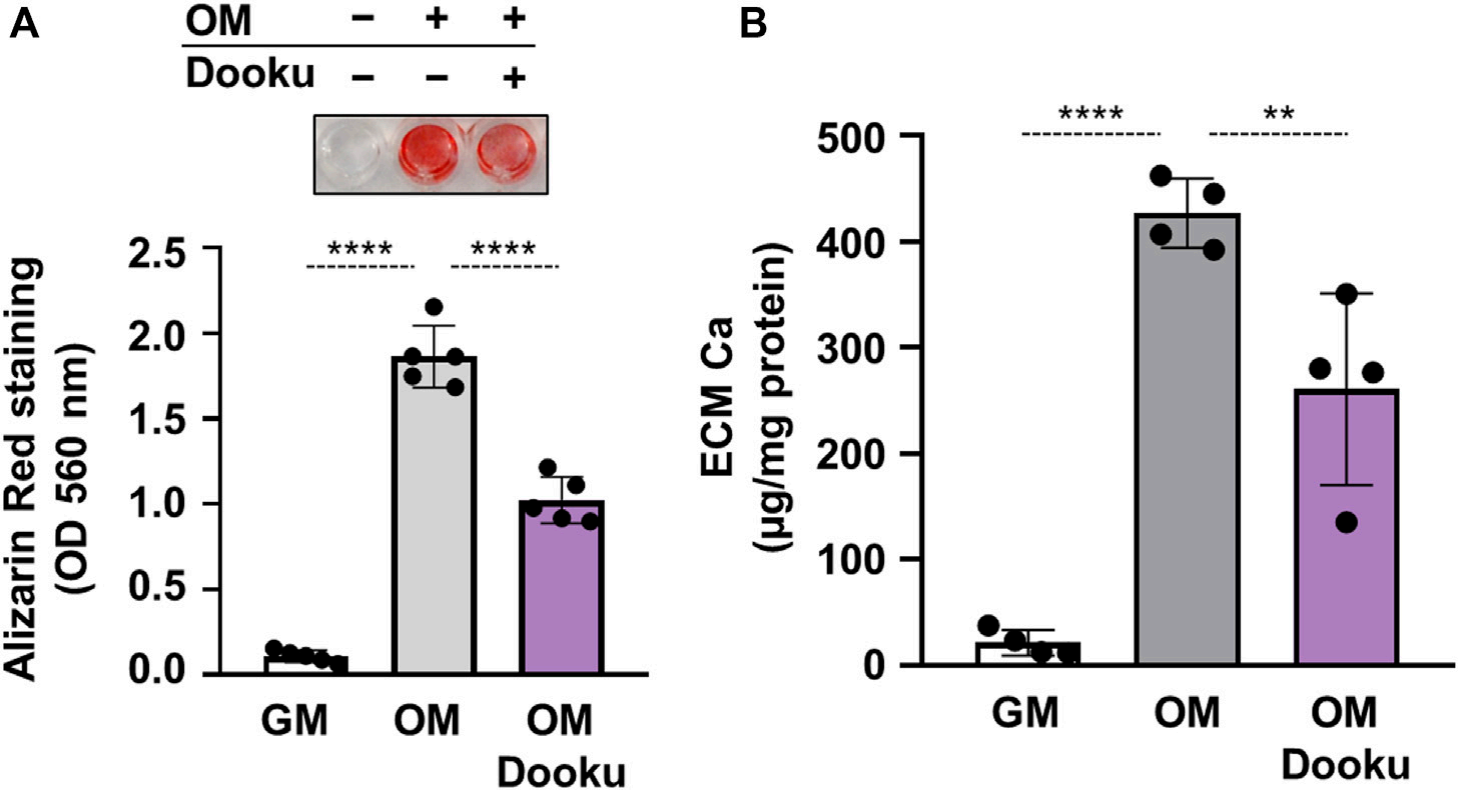
Piezo1 is critical in OM-induced HAoSMCs calcification. **(A,B)** HAoSMCs were exposed to control medium (GM) or OM (excess P_i_: 1.5 mmol/L, excess calcium: 0.6 mmol/L) in the presence of Dooku (5 μmol/L). **(A)** Representative Alizarin Red staining (day 6) and quantification. **(B)** Calcium content of HCl-solubilized ECM (day 6). Data are presented as mean ± SD, *n* = 4–5. **p < 0.01, ****p < 0.001.

Finally, to address the pathophysiological role of Piezo1 in calcification, calcification experiments in the absence of Yoda1 were performed. First, the effect of Dooku1 was tested on OM-induced calcification. As revealed by AR staining and ECM Ca measurement Dooku1 attenuated the OM-induced calcification of HAoSMCs without additional stimulation of Piezo1 channels (Figures 7A,B). Knockdown of Piezo1 by siRNA blocked OM-induced calcification in HAoSMCs as assessed by AR staining, and calcium measurement of ECM (Figures 7C,D).

Taken together, these findings demonstrate the fundamental role of Piezo1 in calcification. Without sufficient expression or function of the channel, administration of the osteogenic medium has no or limited impact on calcification as indicated by AR staining, and ECM calcium content.

## 4 Discussion

Our idea of VC has been transformed fundamentally in the last decades from a picture of a passive degenerative disease to an active, tightly regulated process. The role of SMCs in VC is by now undisputed. The importance of altered intracellular calcium homeostasis both in SMC dysfunction and calcification is also unquestionable. However, the causal relationship between these two phenomena, and the response to how intracellular calcium levels of the SMCs may increase leading to VC, are only partially understood. Our observations provide one of the missing links, revealing a potential source of [Ca^2+^]_i_ rise in HAoSMCs leading to calcification. Furthermore, they also demonstrate that this effect significantly accelerates calcification.

In our study evidence that the Piezo1 channel is involved in arterial medial calcification has been provided. First, Piezo1 expression was confirmed on human aorta rings, and on primary abdominal aortic smooth muscle cells isolated from mice, as well as on HAoSMC cell line. These observations alone were not surprising, since Piezo1 expression has already been detected in several smooth muscle cells (Retailleau et al., 2015; Dalghi et al., 2019) although, to the best of our knowledge, it has not yet been demonstrated on arterial smooth muscle cells of human samples.

Next, both membrane stretch associated with cell swelling induced by hypotonic extracellular solution and pharmacological (Yoda1) stimulations were demonstrated to activate Piezo1 resulting in [Ca^2+^]_i_ rises. However, hypotonicity has been reported to trigger calcium influx through other mechanosensitive channels. Such widely studied plasmalemmal proteins are the members of TRP channels family. The contribution of TRP channels in arterial smooth muscle cells to physiological and pathological processes has not been fully elucidated and is sometimes controversial. Especially, their contribution to AMC has not been studied to date, although the potential expression of about 13 different TRP channels in vascular smooth muscle cells has been suggested in previous studies (Earley and Brayden, 2015). It is also uncertain whether there are different subtypes of some TRP channels in the arterial SMCs of different organs, and whether they are similarly regulated due the physiological stimuli present there, and whether their modulation leads to the same functional result. In most cases their role as the primary receptors of mechanical force is also controversial. However, some observations have reported calcium entry through TRP channels due to increased membrane tension induced by hypoosmotic stress. Although canonical protein channels 5 (TRPC5) expression has not been tested on our HAoSMCs, previous studies have already confirmed their presence in a variety of smooth muscle cell types, including SMCs of human arteries (Yip et al., 2004; Watanabe et al., 2008). Since 240 mOsm has been referred as the activation threshold for TRPC5 in single channel measurements (Shen et al., 2015), in our experiments, where the same level of hypotonicity was applied, TRPC5 contribution to calcium transients should be moderate. Gonzales et al. have reported a stretch-sensitive local signalling network in cerebral artery smooth muscle cells consisting of the phospholipase C γ1 isoform, TRPC6, and TRPM4 (Gonzales et al., 2014). In those experiments 80 mOsm hypotonic treatment was used, which is significantly lower than the one in our experiments. Furthermore, in a recent study by Nikolaev et al., TRP channels heterologously expressed in HEK293T cells were shown to be insensitive to membrane stretch, a physiological stimulus of Piezo channels (Nikolaev et al., 2019). TRP channels did not show inherent mechanosensitivity in this system, but these experiments do not rule out their possible involvement in mechanotransduction. This may be strongly dependent on their relationship to the extracellular matrix, the cytoskeletal and membrane structures. As secondary mechanoreceptors, TRP channels can contribute to the increase of [Ca^2+^] *via* downstream signaling pathway. To elucidate the potential involvement of TRP channels in stretch-induced increases in intracellular calcium levels, SKF 96365, a common TRP channel inhibitor, was used. The significant calcium influx remaining in the presence of the inhibitor indicates activation of other mechanosensitive channels, including Piezo1.

L-type calcium channels expressed in SMCs may contribute to an increased intracellular calcium level, although being voltage sensitive, significant calcium entry cannot be assumed in the absence of depolarization. Work of Retailleau et al. did not reveal any remarkable effect of nifedipine on cytosolic calcium concentration on isolated caudal arteries (Retailleau et al., 2015). Observation of Chen et al. also rule out the role of L-type calcium channels, as treatment of VSMCs with verapamil did not affect intracellular calcium concentration.

Retailleau et al. also described the mechanosensitive properties of the voltage-gated CaV1.2 channels on arterial muscle cells in association with smooth muscle filamin A expression (Retailleau et al., 2016). Although the involvement of CaV1.2 channels cannot be completely ruled out in our measurements, the results of the work just mentioned indicate the importance of calcium influx through these channels at elevated pressure values. Given that Piezo1 responds with exceptional sensitivity to membrane tension relative to other mechanically activated channels (Lewis and Grandl, 2015), we believe that the very low hypoosmotic challenge applied minimizes calcium entry through other channels that are potentially activated by mechanical stimulation.

On the other hand, Yoda1 a small-molecule activator of Piezo1 has no effect either on TRP channels, or on other stretch-activated channels. The increased calcium influx measured using Yoda1 can thus be attributed to the Piezo1 channels. Besides HAoSMC, SMCs isolated from mouse abdominal artery were also examined, and they were also responsive to stimulations of Piezo1, ruling out that the effect seen was the peculiarity of the HAoSMCs. The use of subcultured human aortic smooth muscle cells with a high passage number may pose a risk due to the changed morphological and physiological properties of the aging cells (Bielak-Zmijewska et al., 2014). To mitigate this, we did not use cells with passage number higher than 5.

The Yoda1 analogue, Dooku1, known to reversibly antagonize Yoda1 stimulation of Piezo1, significantly reduced the amplitude of calcium transients on HAoSMCs following Yoda1 treatment (Evans et al., 2018). Under *in vivo* conditions, instead of the transient increase in calcium concentration caused by a single stimulus used in the experiments, repeated stimuli are received as a result of the continuous pressure changes occurring between systole and diastole, accordingly, a permanently high [Ca^2+^]_i_ is formed, which leads to VC. This, in a way, resembles the later calcification experiments, which took place in the presence of Yoda1, thus under permanent stimulation.

Furthermore, we have shown that phosphate-induced calcification of HAoSMCs is associated with an increase in Piezo1 expression level, and accordingly, stimulation of Piezo1 channels resulted in a greater increase in intracellular calcium levels as compared to control cells. These observations clearly indicate that the Piezo1 expression, the intracellular Ca^2+^ level, and the calcification are interrelated. We also pointed out that Yoda1-induced activation of Piezo1 channels significantly enhances calcification on both HAoSMCs and *ex vivo* aorta rings, mentioning the limitation of the experiments, that no mechanical stimulation was used in the case of aorta rings. AR staining, measurement of calcium in the extracellular matrix, and the level of osteocalcin, a marker of osteogenic differentiation of SMCs all support this observation. Both Transglutaminase 2 level, a critical signal for calcification programming in smooth muscle cells, and expression of runt-related transcription factor 2 (Runx2), a master regulator of bone development, which was shown to be crucial for osteogenic phenotype change and mineral deposition in arterial medial calcification were potentiated upon Piezo1 activation (Lin et al., 2015). Moreover, *in vitro* siRNA-mediated gene silencing of Piezo1 on HAoSMCs revealed fundamental role of Piezo1 in calcification. In knockdown cells the osteogenic environment was not able to induce considerable rise neither in AR density, nor in ECM calcium content, while in scrambled cells both parameters were significantly higher in OM complemented with Yoda1 than in GM. These findings suggest that Piezo1 channels are determinants of the calcification process under osteogenic conditions (that can be found *e.g.* in patients with chronic kidney disease) even without stimulation, as in control HAoSMC the osteogenic environment enhanced the values of the two aforementioned parameters, while these changes were absent in knockdown cells.

The central role of both extracellular and intracellular calcium concentrations in calcification have been extensively documented (Yang et al., 2004; Berra-Romani et al., 2008; Rodenbeck et al., 2017). In particular, treatment of VSMCs with BATPA-AM, an intracellular calcium chelator, reduced the rate of calcification (Kapustin et al., 2011). However, to the best of our knowledge, in the growing literature there is no clearly identified mechanism capable of significantly increasing the cytosolic calcium concentration of smooth muscle cells, and, as a consequence, fully explain the process of calcification.

These observations led to the conclusion that calcium is released from internal stores. However, this conclusion needs to be reviewed and supplemented because calcium can also enter the cell from the extracellular space through the Piezo1 channels, as shown in the work described here. This mechanism is also consistent with the finding that extracellular calcium levels play an important role in the mineralization of SMCs (Yang et al., 2004).

The process of VC is analogous to bone formation in many aspects. Both processes involve changes in gene transcription, overproduction of mineralization-regulating proteins, and concomitant release of mineralization-competent matrix vesicles (Anderson et al., 2005; Duer et al., 2008). Elevated intracellular calcium levels have been proposed as a trigger for production of mineralising vesicles in both cases (Kirsch et al., 2000; Kapustin et al., 2011). In bone, retinoic acid has been demonstrated to induce Ca^2+^ influx, and stimulate the release of specialised, annexin-containing, mineralising matrix vesicles by chondrocytes in the epiphyses leading to its proper function: calcification (Iwamoto et al., 1993; Kirsch et al., 2000; Wang and Kirsch, 2002; Wang et al., 2003). It is now appreciated that in vascular SMCs the transformation from non-calcifying to calcifying matrix vesicles is a response for the pathological changes of intracellular calcium level accompanied by suppressed loading with calcification inhibitors (Kapustin et al., 2011). Although a satisfactory explanation for the increasing calcium level that triggers further downstream signaling pathways is still an open question. In a recent work, the increase of [Ca^2+^]_i_ has been ascribed to the release from endoplasmic reticulum through the activation of NADPH Oxidase 1 (NOX1) and mitogen-activated protein kinase (MEK1 and Erk1/2) pathways. However, inhibition of these pathways only partially abolished calcification suggesting the involvement of other possible mechanisms (Chen et al., 2018). Based on our results the calcium influx through Piezo1 channels might be the missing link. It cannot be ruled out that calcium released from intracellular stores contributes to IMC and is triggered by the modification of signaling pathways, while Ca^2+^ entering from the extracellular space contributes to the formation of AMC and is caused by a mechanical effect. Deciding this, however, requires further experiments. Furthermore, the expression of Piezo1 channels is not uniform along the vasculature. Higher expression was detected in resistance arteries and lower expression in conduit arteries, with an increasing trend in the distal direction within each section (Retailleau et al., 2015). Their function can also show regional differences due to, among others, the difference in pressure in the given section (systemic vs pulmonary circulations) (Chen et al., 2022). Therefore, the extent of their contribution to the elevation of intracellular calcium concentration, and so, to the calcification can also vary (Badin et al., 2022). Our observations refer to the systemic circulation, primarily to the section of the large arteries.

In mesenchymal stem cells the expression level of Piezo1 has been proven to correlate with the degree of osteoblast differentiation and the suppression of adipocyte differentiation (Sugimoto et al., 2017). Furthermore, the direct contribution of Piezo1 to mechanical-load-dependent bone formation has also been demonstrated (Sun et al., 2019). Without activation of Piezo1, *i.e.*, in the absence of mechanical loading, both channel expression and osteoblast function are reduced, while physical exertion stimulates both the expression of Piezo1 and osteoblast activity.

Moreover, Piezo1 involvement in hypertensive arterial remodelling has already been characterized (Retailleau et al., 2015), and presented as a key player in arterial mechanotransduction. Increased [Ca^2+^]_i_ has been measured upon channel opening, the potential activation of the Transglutaminase 2 has been proposed as a stimulator of the remodeling of the cytoskeleton and the ECM. The modified structure of ECM, degradation of elastin and increased proportion of collagen is thought to worsen the condition of the blood vessels (Jacob, 2003), and creates favorable conditions for mineralization (Simionescu et al., 2005). Since the force-from-lipid mechanism (Martinac et al., 1990; Teng et al., 2015) appears to be the most likely for gating Piezo1 channels (Cox et al., 2016a; Cox et al., 2016b; Syeda et al., 2016), a local change in the structure of the cytoskeleton and ECM *via* the altered membrane forces leads to alteration in the function of the Piezo1 channels (Gottlieb and Sachs, 2012; Poole et al., 2014; Gaub and Müller, 2017; Cox et al., 2018; Ridone et al., 2019). The consequence might be the increased sensitivity of Piezo1 channels associated to the increased membrane stiffness. Thus, arterial stiffness and AMC mutually reinforce one another, creating a vicious cycle in which VSMCs play a central role (Van den Bergh et al., 2019). Since the calcium influx through the mechanosensitive calcium channel Piezo1 seems to be an essential step in this cycle, it could explain the mechanism by which VSMCs detect and respond to current matrix stiffness.

It is arguable, of course, to what extent the activation of the Piezo1 channel by Yoda1 can be considered a physiological/pathophysiological stimulus. Nevertheless, the use of a specific agonist provides a clear environment for studying the consequences of channel activation. In addition, the fact that the use of the Piezo1 inhibitor Dooku1 alone, without activating the Piezo1 channel, reduces the rate of calcification confirms our hypothesis that the Piezo1 channel is an active participant in calcium homeostasis under physiological conditions and is a major contributor to AMC formation. Although Dooku1 was originally documented as an inhibitor of Yoda1-induced Piezo1 channel activity, our experiments also suggest that constitutively active Piezo1 channels are expressed on HAoSMCs, and the inhibitor has an effect on constitutively active Piezo1 channels as well, as demonstrated in recent studies on perivascular adipose tissue and odontoblasts (Matsunaga et al., 2021; Miron et al., 2022).

Our findings that the Piezo1 channel has remarkable impact on the regulation of cytosolic calcium levels in VSMCs and thus it is a determinant in the formation and progression of calcification may explain the hitherto unclear question of what leads to a significant increase in intracellular calcium concentration in VSMCs, which might be able to stimulate the further steps of mineralization studied extensively earlier. Based on the role of the Piezo1 channel revealed here, they may be a potential therapeutic target that could prevent or slow the progression of AMC.

## Data Availability

The raw data supporting the conclusions of this article will be made available by the authors, without undue reservation.
